# Enhancing research informatics core user satisfaction through agile practices

**DOI:** 10.1093/jamiaopen/ooab103

**Published:** 2021-11-30

**Authors:** Andrew R Post, Jared Luther, J Maxwell Loveless, Melanie Ward, Shirleen Hewitt

**Affiliations:** 1 Research Informatics Shared Resource, Huntsman Cancer Institute, University of Utah, Salt Lake City, Utah, USA; 2 Department of Biomedical Informatics, University of Utah, Salt Lake City, Utah, USA; 3 Research Administration, Huntsman Cancer Institute, University of Utah, Salt Lake City, Utah, USA

**Keywords:** clinical research informatics, core facilities, software engineering, agile methodology, survey, quality improvement

## Abstract

**Objective:**

The Huntsman Cancer Institute Research Informatics Shared Resource (RISR), a software and database development core facility, sought to address a lack of published operational best practices for research informatics cores. It aimed to use those insights to enhance effectiveness after an increase in team size from 20 to 31 full-time equivalents coincided with a reduction in user satisfaction.

**Materials and Methods:**

RISR migrated from a water-scrum-fall model of software development to agile software development practices, which emphasize iteration and collaboration. RISR’s agile implementation emphasizes the product owner role, which is responsible for user engagement and may be particularly valuable in software development that requires close engagement with users like in science.

**Results:**

All RISR’s software development teams implemented agile practices in early 2020. All project teams are led by a product owner who serves as the voice of the user on the development team. Annual user survey scores for service quality and turnaround time recorded 9 months after implementation increased by 17% and 11%, respectively.

**Discussion:**

RISR is illustrative of the increasing size of research informatics cores and the need to identify best practices for maintaining high effectiveness. Agile practices may address concerns about the fit of software engineering practices in science. The study had one time point after implementing agile practices and one site, limiting its generalizability.

**Conclusions:**

Agile software development may substantially increase a research informatics core facility’s effectiveness and should be studied further as a potential best practice for how such cores are operated.

## Lay Summary

The Huntsman Cancer Institute Research Informatics Shared Resource (RISR), a software and database development core facility, sought to address a lack of published operational best practices for research informatics cores. It aimed to use those insights to enhance effectiveness after a substantial increase in team size coincided with a reduction in user satisfaction as measured by annual user surveys. We hypothesized that adopting agile software development practices, which have become standard outside of science but have low adoption in science including biomedicine, may address the problem. RISR implemented agile practices for its software and database development operations in 2020, and user surveys later that year suggest that user satisfaction improved. The article describes the potential benefits of agile techniques for software and database development in research informatics cores, RISR’s implementation of those practices, and the survey results. The article discusses limitations and generalizability of the analysis, and it makes recommendations for implementing agile practices. This work may assist similar cores in optimizing their effectiveness, and it may help the informatics community develop operational best practices for research informatics cores.

## INTRODUCTION

Academic medical centers establish core facilities to centralize expertise, software, databases, and equipment for research labs to share.^1–4^ Research informatics cores provide data management and software development services, often as part of National Institutes of Health (NIH)-supported cancer centers^5^ and clinical and translational science institutes.^6^ While these cores historically have operationalized and supported clinical research informatics tools and methods,^7^^,^^8^ today these cores often have a broader scope encompassing data management across the translational spectrum.

While Huntsman Cancer Institute (HCI) has had a research informatics core for more than 25 years, in general research informatics cores are a recent phenomenon. As a result, the literature on how to deliver high quality services is limited. We found literature on success criteria for bioinformatics cores,^9^ which tend to focus on data analysis not management; and governance and sustainability concerns,^10^^,^^11^ which are important but only part of a core’s success. This work aims to identify best practices for a research informatics core’s software and database development and use those insights to enhance our core’s effectiveness and efficiency.

## BACKGROUND AND SIGNIFICANCE

### Software engineering in science

The emergence of research informatics cores reflects the emergence of computer programming as a scientific activity. However, adoption of programming best practices from software engineering^12^ in science is low, leading to sustainability problems, buggy code, insufficient documentation, and poor usability.^13–15^ These concerns impact the quality of science, even for software used only by the researchers who developed it.^14^^,^^15^ The concerns are likely even greater when producing larger and more complex software applications intended for hundreds of users, as core facilities do.

Reasons for limited adoption of software engineering in science include lack of familiarity with the software engineering field and a perception that software engineering practices are a poor fit for science.^16–19^ While traditional waterfall engineering models^12^ envision collecting requirements for a software application entirely in advance of development, it is rare that investigators can fully envision an application in advance because the processes being modeled are complex and incompletely understood. In addition, until recently most software engineering projects were developed by small groups or individuals^20^ who might not have perceived the value of formal software engineering methods that are employed in industry with larger teams.^17^^,^^18^

Newer agile models of application development^12^^,^^21^ may better align with science by prescribing an iterative development approach that does not require software to be specified fully in advance. Evidence suggests that agile approaches benefit a wide variety of organizations, particularly those that operate dynamic environments like in science.^22^ Agile approaches value interactions between team members, keeping software in a working state throughout development, collaborating with customers, and welcoming change. These values are recognizable to most scientists. We believe that software engineering practices like agile that strive to produce more user-focused and reliable software may enable more accurate, rapid, and reproduceable research results.

In agile application development, responsibility for communication between customers and software engineers lies in a role called the product owner.^22^ Product owners translate users’ requirements, needs, and feedback into a vision for the software that is reflected by a prioritized list of development tasks called the backlog. They manage project scope, deadlines, and budget; negotiate tasks with the software developers; and ensure that the resulting software provides the greatest value for users based on current needs and available resources. In addition, product owners arrange for frequent software demonstrations to obtain user feedback, and they otherwise serve as a proxy for the customer at software developer meetings. Scientific software groups in academia may not sell software and thus not have products in the typical sense, but they do have customers whose needs must be met if their groups are to remain viable.

Increased awareness of these practices in science recently led to the creation of societies that have formulated a research software engineer (RSE) role,^23^^,^^24^ such as the US Research Software Engineer Association (US-RSE)^25^ and similar organizations overseas.^26^ A RSE differs from a software engineer in that career growth involves acquiring scientific expertise on-the-job in addition to pure software development expertise, including writing grants in some cases. A similar role in bioinformatics, the bioinformatic engineer, was recently proposed.^18^ RSEs are envisioned to stay in science their entire careers due to specialized expertise they gain in high performance computing, physics, and other domains that tend to be funded in the US by the National Science Foundation; or bioinformatics funded by the NIH. These scientific disciplines are tech adjacent in the sense that programming has become an essential skill for their researchers.

### Software engineering in research informatics cores

In biomedical science, despite the potential advantages of agile software development, programming guidance in the literature focuses on software engineering tools like version control, testing, and task tracking, which are important but only scratch the surface of current best practices.^15^^,^^27^^,^^28^ We found only passing reference to the product owner concept in the biomedical literature.^20^ Database development, which is common in research informatics cores, has a different culture that has adopted few of the tools of modern software engineering, even the basics like version control, though they likely would have similar benefits.^29^

While there is no literature on adoption of software engineering best practices by biomedical core facilities in general or research informatics cores in particular, adoption is likely as low as in the rest of biomedicine. Most cores are operated by scientists^3^ whose leadership experience and training are in running labs not software engineering groups.^4^ Also, until recently these cores were small like other scientific programming teams. The infrastructure software and databases that cores build have frequently changing requirements due to changing scientific priorities and data management requirements from funding agencies, academic departments, and faculty. As a result, cores would likely benefit from far greater adoption of agile techniques.

In fact, the customer population of research informatics cores suggests an even stronger need for the potential communication benefits of agile techniques than elsewhere in science. The NIH-funded investigators in medical, population health, and other disciplines that these cores serve usually have no programming background. Similarly, these cores’ software engineers typically have no graduate-level medical or scientific research training.^18^^,^^30^ Software engineers in a research informatics core, in our experience, tend to have previous jobs outside of science and medicine, and their next jobs are usually outside of science and medicine, making them different than the RSEs above. Scientific computing in research informatics cores is like industry software development in these respects.

The different customer population and staff composition suggest that research informatics cores are a special case in scientific computing. Putting product owner responsibilities into a dedicated information conduit role, rather than expecting technical staff to interact with customers informally alongside their other responsibilities, may increase the likelihood of strong customer relationships and successful software development. In addition, the product owner role may enable research informatics cores to provide leadership to their institutions in creating the strong relationships between researchers and technical disciplines that are needed to advance modern biomedical science.

### HCI Research Informatics Shared Resource

**Figure 1.**
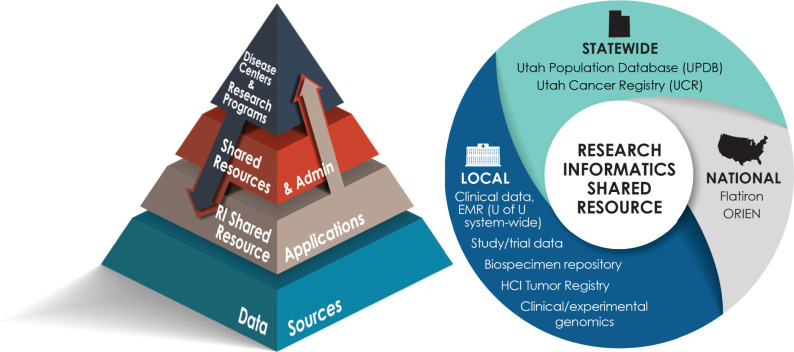
Pyramid on the left showing that RISR serves as a conduit of research data to the other cancer center shared resources, as well as the cancer center research programs and disease centers (teams of clinician-researchers). Circle on the right illustrates the breadth of data managed by RISR. ORIEN, Oncology Research Information Exchange Network.^33^^,^^34^

The Research Informatics Shared Resource (RISR)^31^ has built over 30 home-grown software applications and databases serving researchers and cancer center research administrators. As shown in Figure 1, RISR serves as the conduit for cancer center data for the other HCI cores, which include bioinformatics, high-throughput sequencing,^32^ biostatistics, genetic counseling, the biorepository, and more. RISR also serves data to the cancer center’s research program members, other faculty conducting cancer-focused research, and cancer center-wide initiatives. RISR’s database support spans the entire translational spectrum. RISR is directed by a MD-PhD scientist (ARP) with a substantial software development background. Its staff are almost all software engineers and data analysts, most of whom do not have graduate-level training in the biological sciences or medicine.

Historically, RISR software applications were built by individual engineers, or in a few projects by teams with a technical lead, according to the waterfall model. RISR adopted basic software engineering tools like version control and electronic task tracking many years ago. Some teams partially adopted agile processes including frequent short status meetings called scrums, led by a senior scrum master and agile coach (JL). Engineers shared responsibility for communicating with users, and they did so in an informal fashion that varied in frequency and methods from project to project. This hybrid adoption of agile and traditional practices is common and sometimes called the water-scrum-fall model.^35^ It was effective for many years as indicated by annual user surveys measuring quality of service and turnaround time.

However, in 2016–2017, RISR grew from 20 to 31 FTEs due to the launch of a large new project that also pulled some existing staff from other projects. Afterward, annual HCI user survey scores fell. While decreases in user satisfaction may be partially explained by slower progress on these other projects, anecdotal comments from RISR users suggested that the increase in size may have interrupted relationships that RISR had with its customers. We concluded that, at a team size of 31, previous informal methods of interacting with users were no longer effective, and RISR needed to be more systematic about how it engages its user community.

### Objective

Starting in January 2020, RISR adopted agile practices more completely. It retrained staff in agile approaches and mandated the use of agile application development for all project teams. Small projects developed by individuals were aggregated into related project groups developed by teams. These teams implemented scrums, dividing work into 3- to 4-week “sprints”; meetings for sprint planning; and meetings for continuously prioritizing (grooming) the backlog. In addition, the core director appointed a product owner for each team. We hypothesized that these agile practices may substantially enhance investigator satisfaction with a core’s services. Below we describe RISR’s agile implementation in greater detail, and we report RISR’s user survey scores from late 2020 that provide insight into the implementation’s impact.

## MATERIALS AND METHODS

**Figure 2.**
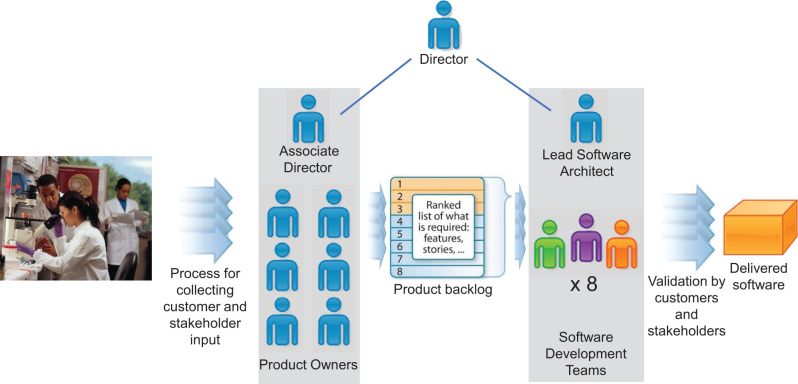
Structure of RISR. Product owners on the left, governed by the associate director, serve as a conduit for collecting customer and stakeholder input and translating that input into a product backlog to guide the software development teams. The product owners also guide software validation by customers and stakeholders, and they make decisions to release software. RISR currently has 6 product owners and 8 software development teams of various sizes.

RISR’s revised structure is illustrated in Figure 2. All personnel report to the core director. The associate director of the core governs product owner activities (on the left side of Figure 2) and is responsible for developing overall product owner strategy. A chief software architect governs software engineering activities (on the right side of Figure 2); oversees software development strategy and processes; and develops overall software architecture. An agile coach and scrum master governs agile software development practices and runs scrum meetings, sprint planning, and backlog grooming meetings.

In addition to governing product owner strategy, the associate director provides aspects of traditional project management like coordinating product roadmaps for communication with other RISR teams and external stakeholders. However, in keeping with an iterative agile approach, product roadmaps only make commitments for the current and next quarters. Future work is captured in roadmaps with high level descriptions and no committed timeline to avoid overcommitting and knowing that stakeholders are likely to reprioritize and rethink future work as needs change.

Product owners collect customer and stakeholder input and communicate it to technical staff as user stories that describe the type of user, the goal or objective, and the benefit or value. They record user stories in the backlog (Figure 2), prioritize them, and review them with the software developers. In addition, product owners validate the software to ensure that it does what the user stories describe prior to releasing it to users. As an extension to agile practices, larger projects have “vision” meetings in which the core director and RISR’s agile coach support the product owner in prioritizing user stories.

To facilitate communication between product owners and development teams, each team has a technical lead who is responsible for implementing the customers’ vision for the software as articulated by the product owner. Technical leads also coordinate with the core’s chief software architect to ensure that the project’s code conforms to software development best practices.

## RESULTS

In January 2020, the RISR director appointed 5 product owners from existing staff. The agile coach provided training as described above. In July, HCI appointed one of the product owners (SH) the associate director (Figure 2). Also, in mid-2020, RISR hired a new business analyst who serves as a product owner, and appointed a product owner from existing staff, for a total of 6 product owners. The product owners are all part-time in that role. Their other roles include software engineer (1), data architect (1), software architect (1), business data analyst (2), and scrum master (1). For the project in which RISR’s scrum master serves as product owner, the project’s technical lead serves as scrum master.

RISR used HCI’s annual user survey of its shared resources to evaluate the impact of RISR’s new structure in its first year. The survey is administered by the HCI Research Administration office and is distributed through Survey Monkey to cancer center members and recent users of at least one HCI shared resource. While the survey asks many questions that applied to RISR, the questions that are the focus of this analysis are listed in Table 1.

**Table 1.** 2013–2020 shared resource user survey questions about the quality and turnaround time of services provided by RISR

Question	Possible responses
Overall, how would you rate the quality of the service/product you received from the Research Informatics Shared Resource?	Exceptional High Average Poor Unacceptable
Overall, how would you rate the turnaround time for receiving data, products, or other services from the Research Informatics Shared Resource?	Exceptional High Average Poor Unacceptable

**Figure 3.**
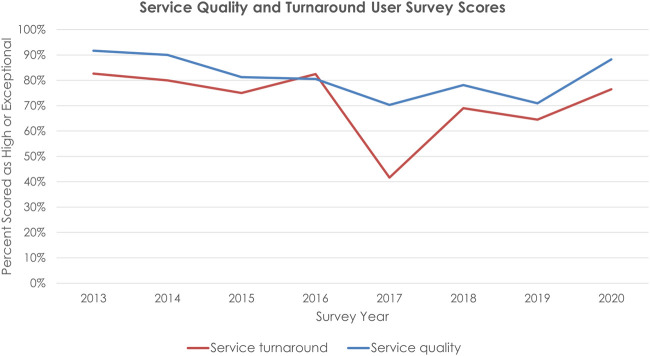
Service quality and turnaround user survey scores, 2013–2020. Number of respondents per year: 24 in 2013, 20 in 2014, 16 in 2015, 41 in 2016, 37 in 2017, 32 in 2018, 31 in 2019, and 17 in 2020.

The user survey was open between September 11 and September 24. Thus, it provided feedback 9 months after RISR introduced agile practices into its operations. A total of 17 respondents answered the questions (Table 1) out of 52 identified RISR users (33% response rate). For quality of service, 7 answered exceptional, and 8 answered high, for a total of 88% who rated quality of service as high or exceptional. For turnaround time, 7 answered exceptional and 6 answered high, for a total of 76% who rated turnaround time as high or exceptional. The same questions were posed to users in the 2013–2019 surveys, and trends for 2013–2020 are shown in Figure 3. Compared to 2019, survey scores rose in 2020 by 17% for quality and 11% for turnaround time. Response rate in 2019 was 58%. Scores between 2017 and 2019 were all lower than the 2020 scores.

## DISCUSSION

RISR (Figure 1) increased its adoption of agile software development in early 2020 after an increase in FTE count co-occurred with decreases in service and turnaround scores (Figure 3) in annual user surveys. As part of this change, RISR implemented a product owner team (Figure 2) under the rationale that the core’s growth necessitated more systematic user engagement than in the past. While commercial software engineering teams have included product owners for years, we are unaware of other core facilities that have created a product owner team. In addition, while many publications have articulated the theoretical benefits of agile application development in science,^20^^,^^30^^,^^36^^,^^37^ this is the first study we could find that has quantified the end-user experience benefits of adopting it.

In theory, agile application development increases the likelihood that the right software is developed at the right time, resulting in faster software development due to less “unnecessary” work taking place. Agile is also designed to facilitate user engagement, which is critical in science because software engineers often do not possess deep biomedical science knowledge.^18^^,^^30^ While increasing speed of development may increase user satisfaction, keeping users engaged in software development progress may increase satisfaction independently of speed. We believe that the most likely explanation for the observed increase in user ratings of service and turnaround time is the increase in user engagement via product owners. Measuring velocity change in RISR’s software development teams is future work. In addition, detailed measurement of user engagement may be a fruitful future path for better understanding the impact of agile approaches in science.

Other potential interpretations include increases in the number and complexity of the projects that were conducted during the years of lower survey scores, followed by a return to a smaller number of projects of lower complexity. However, during 2020 the project that was launched in 2016 was still ongoing, and many large projects were launched including full rewrites of the front-end code of 17 of the core’s software applications. If anything, the core’s workload was higher in 2020 than it was when the survey scores dropped. Another potential explanation is turnover of stakeholders and investigators who were particularly critical of the core, but stakeholders of major projects remained the same throughout the years analyzed. The survey did not attempt to discern whether a low score from an investigator might be due to a single bad experience shortly before getting the survey request versus consistently negative impressions over time.

RISR achieved these benefits within 9 months of introducing product owners despite limitations in the implementation of its product owner team. RISR’s product owner team lacks full-time product owners, which is the norm in commercial teams. For a project with many software engineers, collecting and managing user stories, creating and maintaining the product vision, and communicating with users and software engineers is ideally a full-time job. In addition, giving product owner roles to existing staff decreased software engineering capacity. As is typical in academia, RISR staff often have multiple responsibilities, even with a staff size of 31. Our results suggest that a successful product owner implementation is possible despite the resource constraints that are typical in academia.

The survey data has multiple limitations. The survey was used retrospectively rather than constructed for the needs of this study, it included questions about HCI’s other shared resources, and it was sent to a large group of investigators, not just those to whom RISR provided support.

In addition, while the response rates are not unusual for this type of survey, the absolute response counts were small. RISR’s overall user count as measured by software logins or a clinical trial managed by RISR’s software (142 cancer center members in 2019) was substantially higher than the number of investigators who requested services (48 in 2018, 53 in 2019, and 52 in 2020). The latter was the survey’s denominator for consistency with measurements from HCI’s other shared resources. Higher response counts (Figure 3) were associated with lower user satisfaction scores, suggesting that researchers were more likely to make time for the survey when they were unhappy with the service.

Run charts, like in Figure 3, are a common practice in healthcare quality improvement to detect early signals of improvement after process change.^38^^,^^39^ This work was an informatics quality improvement activity, and the goal was to obtain an early signal of whether agile practices had an effect. Unlike traditional analyses that aim to determine statistical significance, the run chart preserves the time order of the data, which is useful for decision-making on whether to make a process change permanent. There are quantitative process control methods for determining whether an improvement is nonrandom. However, a minimum of four more years of survey data would be necessary to detect a shift (data consistently above the baseline median) or trend (data consistently increasing), and two more years of data to detect a run (nonrandom crossings of the baseline median line),^39^ which would provide useful information but not as an early signal. Informal feedback from users since the 2020 survey indicates that there was indeed a noticeable improvement in service, and the difference is also valuable for reporting on performance for HCI’s Cancer Center Support Grant. Future surveys might be helpful in determining whether the improvement is nonrandom, but core facilities like RISR constantly adjust operations to improve service, thus introducing confounders.

Despite the limitations of the survey, we believe that these user satisfaction improvements are likely to be repeatable by other core facilities that adopt agile application development. In rapidly changing scientific fields, the waterfall model may lead to infrastructure software that is outdated by the time that it is released. Increased user engagement is especially likely to increase software quality in scientific domains where the software engineering staff may lack domain knowledge that must be provided by users. These benefits are likely to be greater the more products the core produces due to increased likelihood that user communication might otherwise fall through the cracks. As a result, agile software development may be even more valuable to cores than it is to research laboratories.

Furthermore, based on RISR’s experience and the literature, agile practices may have the most impact on larger cores that have multiperson teams. Because agile practices originated in the software engineering community,^21^ such cores have an opportunity to adopt agile practices themselves, and then provide a service to their institutions in agile coaching for other shared resources and research labs. Recommendations for those that wish to adopt agile approaches include:

1. Understand that adopting agile is a multiyear process that involves continuous refinement. Agile requires training, behavioral change, and appropriate information technologies. The water-scrum-fall model is frequently an intermediate step. At RISR, the overall process had begun prior to 2020, and it continues to undergo refinement.
2. Hire expertise like a certified scrum master and agile coach, which RISR did. The meeting volume associated with managing the backlog, sprint planning, and scrums is substantial, and these meetings must be efficient and effective to retain staff buy-in. RISR plans to budget for an administrative assistant to manage agile-related meeting schedules.
3. Allow product owners to focus at least 50% effort on product owner duties. The nature of the other 50% of their duties can vary depending upon expertise. For projects with 5 or more engineers, product ownership is ideally a full-time job.
4. Empower teams to self-organize in estimating and assigning work, while holding them accountable for results. Achieving this may require a culture change in organizations that are used to work being assigned in a command-and-control fashion.
5. Customize agile methods for your teams and consider complementary approaches that may be better suited to some aspects of the teams’ work. Complementary approaches are particularly useful when working with other organizational units that have traditional project structures. For example, traditional project management may be valuable in clarifying expectations and timelines when asking user groups to make time for training and application testing.

## CONCLUSION

Data from one research informatics core suggest that introduction of agile practices can measurably and positively impact user perceptions of quality and turnaround time. It is likely that an expanded study will show that agile approaches have the most impact on larger informatics core facilities and cores that need to be more responsive to user needs. These findings may assist the leadership of similar cores in optimizing their effectiveness. They also may provide guidance to the informatics community in developing operational best practices for research informatics cores.

## FUNDING

Research reported in this publication utilized the Research Informatics Shared Resource at Huntsman Cancer Institute at the University of Utah and was supported by the National Cancer Institute of the National Institutes of Health under Award Number P30CA042014. The content is solely the responsibility of the authors and does not necessarily represent the official views of the NIH.

## AUTHOR CONTRIBUTIONS

ARP, SH, and JL implemented agile software engineering practices at RISR. JML and MW led administration of the annual user surveys and provided substantial input on their use in this manuscript. ARP and SH analyzed the survey results. ARP drafted the manuscript, and JL, JML, MW, and SH reviewed it and made edits.
